# Provider-perspective cost analysis of comprehensive pediatric cataract management in a low-income setting

**DOI:** 10.1038/s41598-025-32417-9

**Published:** 2026-01-19

**Authors:** Broder Poschkamp, Rainald Duerksen, Motema Malemo Jaspe, Edith Mukwanseke, Andreas Stahl, Rudolf Guthoff, Astrid Moanda, Ellen Catrin Steinau, Janvier Kilangalanga Ngoy, Steffen Fleßa

**Affiliations:** 1 Department of Ophthalmology, University Medicine Greifswald, Ferdinand-Sauerbruch-Street, 17475 Greifswald, Germany; 2 Fondation Vision RDC, Lassa avenue 09B, Mazal, Kinshasa/MontNgafula, Democratic Republic of the Congo; 3 St. Joseph Hospital Kinshasa, Democratic Republic of the Congo, Kinshasa, Democratic Republic of the Congo; 4 Institute for Biomedical Engineering, Rostock University Medical Center, Friedrich-Barnewitz-Street 4, 18119 Rostock, Germany; 5 Réhabilitation à Assise Communautaire (RAC/CBR), P.O. Box 322, Kinshasa, Democratic Republic of the Congo; 6 Department of Gynaecology, University Medicine Greifswald, Ferdinand- Sauerbruch-Street, 17475 Greifswald, Germany; 7 Department of Healthcare Management, University of Greifswald, Friedrich-Loeffler-Straße 70, 17487 Greifswald, Germany

**Keywords:** Pediatric cataract, Cataract surgery cost, Screening programs, Cost analysis, Democratic republic of the congo, Diseases, Health care, Medical research

## Abstract

Childhood cataract is a leading cause of avoidable blindness in sub-Saharan Africa, but affected children rarely self-present. Therefore, programs must fund proactive case-finding and ongoing follow-up. We quantified, from a provider perspective, the costs of identification, surgery, follow-up, and rehabilitation in Kinshasa, DRC. We analyzed costs (March 2024) of a community-embedded program linked with hospital services. The pathway from screening to bilateral surgery and two-year follow-up was mapped. Costs (2024 USD) from hospital and payroll data and interviews were cross-checked regionally. A step-fixed model captured screening expansion; patient-incurred costs were excluded. The network held 32 screenings for ~ 134,400 people annually, identifying 38 children (28.3/100,000). Unit costs were $0.08 per person screened and $268.4 ± 53.7 per child (30.7%) identified; surgery $529 per child (60.5%); follow-up $77 ± 15.4 (8.8%). Average continuum cost was $875 ± 69.1 per child. Treating 38 children cost $33,245 ± 2,625.2 annually. Scaling to 1,000,000 people required eight teams, identifying 283 children at a program cost of $253,223 ± 20,678. Comprehensive pediatric cataract care is feasible at ~$875 per child when budgets include community case-finding, coordination, and follow-up alongside consumables and theatre. Volunteer-reliant case-finding and rehabilitation require funded core staff for stability. These data provide a pragmatic template for scaling programs in low-resource settings.

**Supplementary Information:**

The online version contains supplementary material available at 10.1038/s41598-025-32417-9.

## Introduction

Worldwide it is estimated that 1.4 million children aged 0–14 are blind^1^. In sub-Saharan Africa (SSA) alone, as many as 82,000 children live with cataract-related blindness, with about 19,000 new cases arising each year^2^. Without timely intervention, these cataracts lead to lifelong blindness, resulting in lost educational and economic opportunities and a heavy social burden on children and their families^3,4^. This substantial disease burden has made pediatric cataract a priority within global blindness prevention initiatives such as VISION 2020, yet access to effective care remains limited in many low-resource settings^5^.

A critical challenge in addressing childhood cataract is that affected children with their parents rarely self-present for care^6^. Unlike adults who may seek help when vision deteriorates, young children often do not report vision loss, and families may not recognize the problem or know that treatment is possible^7,8^. This underscores the need for proactive case-finding strategies. Effective programs actively engage the community to identify and refer blind children, rather than waiting for families to seek care^2,9,10^.

Comprehensive management of childhood cataract requires an integrated care continuum from patient identification, surgical treatment, follow-up and rehabilitation^11^.While the need for comprehensive pediatric cataract programs is clear, there is little data on the resources and costs required to implement the full spectrum of care in low-income settings. Most existing studies have examined isolated components of care (primarily the surgical episode) rather than the entire care pathway. For instance, a prior analysis at two child eye health tertiary facilities in Africa estimated the direct cost of pediatric cataract surgery at roughly $200–$300 per case (2011 US dollar)^9^. However, that estimate focused only on expenses at the hospital (e.g. surgical supplies, equipment, and personnel for the procedure itself) and did not capture the costs of community outreach, patient identification, or post-surgical follow-up. As a result, health planners and policymakers lack comprehensive evidence on what it truly costs to deliver effective pediatric cataract services from identification to rehabilitation.

In order to bridge this knowledge gap, the present study provides a detailed costing analysis of a pediatric cataract management program in Kinshasa, DRC, covering the entire continuum from case finding to surgery to long-term follow-up. By drawing on a real-world implementation example, we aim to clarify the magnitude and types of resources needed to care for children with cataract in a low-resource setting. This information is intended to inform program planning, resource allocation, and advocacy efforts by illustrating the full investment required to prevent childhood blindness due to cataract in sub-Saharan Africa.

## Methods

### Costing analysis

This study is a cost analysis of a pediatric cataract management program in Kinshasa, Democratic Republic of the Congo, conducted in March 2024. Data were collected through structured interviews with key stakeholders involved in the care continuum, including volunteers, parents of patients, ophthalmologists, anesthesiologists, pediatricians, nurses, social workers, and representatives from community-based organizations. The interview guide was developed by the study team based on prior pediatric cataract literature and piloted with two staff members, after which minor wording changes were made for clarity. Interviews were conducted in English, French, or Lingala by a trained local researcher and were summarized in structured notes that were used to validate and complement the hospital and program cost data. The English version of the interview guide is available in the Supplement. To ensure a comprehensive understanding of the cost drivers, we directly followed the care pathway from community-level screening and identification through hospital-based surgery and postoperative follow-up.

Costs were documented from the provider perspective and include fixed and variable components of the screening infrastructure, hospital services, and human resources. All listed prices (e.g., for surgery, anesthesia, materials, consultations, and hospitalization) were extracted from hospital bills and verified through cross-checks with other regional hospitals to ensure plausibility and local accuracy. Reported salary structures for staff were assessed through both hospital payroll data and interview validation. To characterize parameter uncertainty, we varied staff salaries and follow-up visit unit costs by ± 20%, while retaining observed surgical unit costs from this center, the only facility in Kinshasa providing pediatric cataract surgery, which were consistent with regional adult cataract tariffs and reported price structures.

The costing approach aimed to include all relevant programmatic expenses (e.g., staff salaries, medical consumables, facility usage, follow-up consultations) excluding patient-incurred costs such as transportation or lost income, which were considered too heterogeneous to quantify reliably. All costs are reported in US dollars (2024 USD) and reflect the local healthcare and salary structures applicable during the data collection period.

To model the cost dynamics of scaling the pediatric cataract program, we applied a semi-fixed (step-fixed) costing approach, in which screening costs remain constant up to the capacity of one team (covering approximately 134,400 individuals per year) and then increase discretely when an additional team is required, while treatment costs (surgery and follow-up) scale linearly with the number of children identified^12^. The simulation, implemented in Python 3.8.5 (Python Software foundation), iterated over population sizes from 0 to 1,000,000, calculating total and per-child program costs.

To ensure transparency and reproducibility, we assessed this study against the Consolidated Health Economic Evaluation Reporting Standards (CHEERS 2022) checklist, with the completed checklist provided in Supplementary Table S1. The study was conducted according to the guidelines of the Declaration of Helsinki and approved by the Ethics Committee of the Ministry of Higher Education and Universities (DRC, Kinshasa, No.: ESP/CE/93B/2022), 13 July 2022. Written informed consent was obtained from the legal guardians of all participants in the overarching clinical study, and the data used for this cost analysis were anonymized and aggregated, containing no patient-identifiable information. Patients and members of the public were not involved in the design or conduct of this research.

### Setting and care pathway

Pediatric cataract care in this program is delivered through a partnership between a tertiary eye unit and a community-based rehabilitation and screening organization. Direct medical costs for surgery and scheduled follow-up visits for enrolled children are covered by program funds (a mix of institutional, NGO, and external donor support) and are not charged to families, although our costing uses the underlying hospital tariffs and salary scales as the basis for provider costs. To date, there is no specific governmental funding mechanism for bilateral pediatric cataract care in this setting.

The care pathway starts with training parish-linked volunteers to recognize visual impairment, counsel families, and refer suspected cases to an ophthalmologist for eligibility confirmation. Once a child has been diagnosed with bilateral cataract, a comprehensive preoperative assessment is conducted. This includes a full ophthalmic examination, visual acuity testing, slit lamp examination, fundoscopy, and intraocular pressure measurement, as well as consultations with a pediatrician and an anesthesiologist to identify comorbidities and assess surgical fitness. Because of local constraints, both eyes are operated on in a single session under general anesthesia, with a full change of surgical materials between eyes. Two operating rooms run in parallel, staffed by a surgical team of five, including one ophthalmologist, and a separate anesthesia team consisting of an anesthesiologist and an assistant. Intraoperative biometry is used to determine the appropriate type and power of the intraocular lenses to be implanted. Following surgery, children typically remain hospitalized for seven days, accompanied by a parent or volunteer, during which they receive meals and basic care to support postoperative recovery.

Follow-up visits are scheduled at one, two and four weeks, two, three and six months, one and two years. Community workers and case managers support adherence, refractive correction and amblyopia therapy, and verify school reintegration and glasses use. A more detailed process description can be found in the supplement (Supplement Fig. S1).

### Community-based screening and rehabilitation infrastructure

The screening infrastructure leverages a broad, community-based network coordinated by RAC (Accompagnement des Malades à Domicile, Réhabilitation à base Communautaire, AMD/RAC), working closely with local parishes, community volunteers, and various partnering organizations. The screening and rehabilitation service focuses on education and awareness, screening, guiding individuals toward medical treatment, and supporting social integration. It addresses visual, auditory, and mobility impairments, as well as developmental or intellectual disabilities and other complex neurological or psychiatric conditions (e.g. cerebral palsy, epilepsy syndromes, trisomy 21, and severe trauma-related disorders). The visual impairment team consists of one ophthalmic nurse, one case manager, one social worker, about 20 volunteers for screening, and 12 volunteers for follow-up (Supplement Table 2).

The screening system is built around a hierarchical structure starting at the parish level. There are 218 parishes, each encompassing approximately 10 communities, and every community consists of about 50 to 70 families. With an average of seven people per family, one community has roughly 350 to 490 individuals, and using a midpoint of 60 families, around 420 individuals per community. Ten communities, therefore, amount to approximately 4,200 people per parish, and all 218 parishes collectively encompass around 915,600 individuals. This layered approach, from families to communities to parishes, ensures that the screenings can reach a wide population (Supplement Fig. S2).

## Results

### Community-based screening

Building on this hierarchical, parish-based network, trained volunteers and community workers carry out awareness campaigns and mobilize those who may need medical attention, including children with potential cataracts. Over the course of one year, the program conducted 32 structured screening events, reaching a total of approximately 134,400 individuals. These screenings were based on a hierarchical network model involving 0.62 parishes screened per week, each parish comprising around 4,200 people and about 1,180 individuals come to these screening days and get screened (involves patients with complaints and without), involving at least two trained volunteers per screening session.

**Fig. 1:**
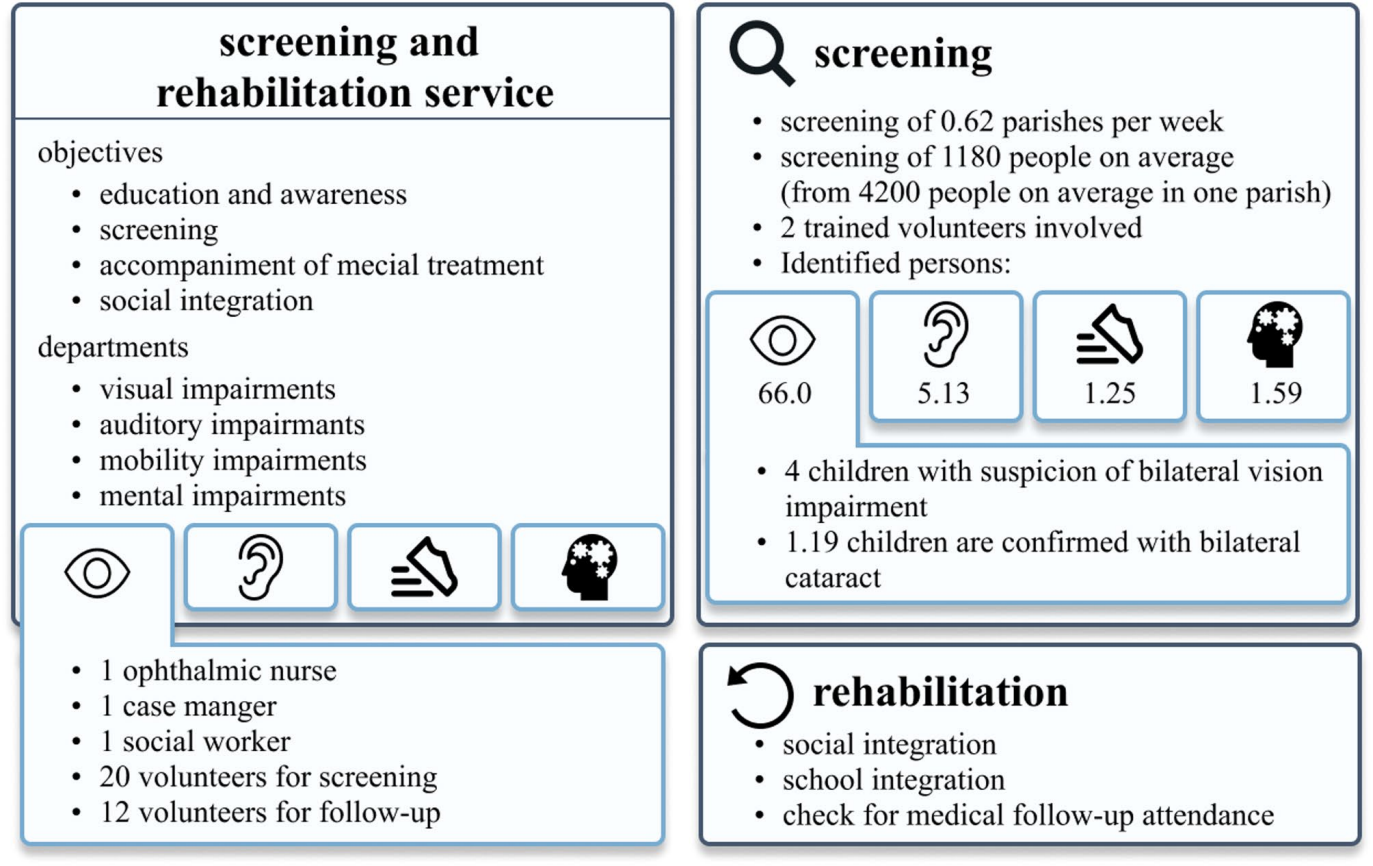
Screening and rehabilitation service information.

Of those identified with potential health problems, the majority have visual impairments (66.0 children), outnumbering auditory (5.1 children), mobility (1.3 children), or developmental/intellectual and other complex neurological or psychiatric conditions (1.6 children). Of these 66 children on average on one screening day, four children (4.0) might present with suspicion of bilateral vision impairment, and 1.19 of these children will be confirmed to have bilateral cataract. In one year, 38 children were identified with bilateral congenital cataract and subsequently referred for treatment. The calculated prevalence of bilateral cataract in this screening situation adds up to 28.3 per 100,000 persons in Kinshasa. After diagnosis, rehabilitation efforts include not only medical follow-ups but also measures for social and school integration, ensuring that improved vision translates into enhanced quality of life and better opportunities for affected children (Fig. 1).

The total annual program for the visual part of the screening cost amounted to $10,200 ± 2,040, primarily covering salaries for an ophthalmic nurse, a case manager, and a social worker. This resulted in a unit cost of $318.75 ± 63.8 per screening, $0.076 per individual screened, and $268.42 per child identified with bilateral cataract (Table 1).

**Table 1:** Activity output and unit cost calculation.

Metric	Value	Explanation
Total number of screenings	32 screenings	Conducted across the year
Total individuals screened	134,400 persons	Based on ~ 4,200 persons per parish × 0.62 parishes/week × 52 weeks
Children identified with bilateral cataract	38 children	Confirmed cases after referral and diagnosis
Unit cost per screening	$318.75 ± 63.8	$10,200 ÷ 32 screenings
Cost per person screened	$0.076 ± 0.015	$10,200 ÷ 134,400 individuals
Cost per child identified	$268.42 ± 53.7	$10,200 ÷ 38 children

### Preoperative assessment and surgical intervention

Preparation for pediatric cataract surgery in Kinshasa involves a careful sequence of steps. According to local ophthalmologists, the Democratic Republic of the Congo has only about 130 ophthalmologists, 80% of whom practice in Kinshasa, and access to specialized care is limited. Of these 130, just 48 regularly perform surgeries. This scarcity of trained surgeons and specialists influences how surgeries are scheduled and planned.

The total cost for pediatric cataract surgery in general anesthesia on both eyes during one hospitalization over 7 days comes to about $529.44. Within the surgical cost structure which makes around 58% of the total cost, approximately 26.18% covers the ophthalmic procedure, 39.08% is attributed to anesthesia, and 34.74% is allocated to surgical materials (detailed in the supplement). The costs are summarized in Table 2.

**Table 2:** Cost and cost centers for pediatric bilateral cataract surgery in Kinshasa (for 2 eyes).

Cost Category	Cost (USD)	Share of total cost (%)	Explanation 2 eyes in one procedure and one hospital visit
Administrative	$31.26	5.9%	Includes proof of identity, admin fees, and documentation
Pre- & Post-surgical Consultations	$119.19	22.51%	Includes first ophthalmic consultation, general examination, pediatric and anesthesia consults
Surgery	$307.04	58.0%	Ophthalmic procedure, anesthesia, and materials
- Ophthalmic Procedure	$80.37	26.18% of surgery share	Direct surgical intervention by the ophthalmologist for 2 eyes
- Anesthesia	$120.00	39.08% of surgery share	Includes anesthesiologist and assistant
- Surgical Materials	$106.67	34.74% of surgery share	Includes lenses, instruments, consumables for 2 eyes
Hospitalization	$56.77	10.72%	a 7-day stay with caregiver, meals, post-op monitoring (8.11$ per day)
Maintenance (non-clinical)	$15.19	2.87%	Includes indirect support services and facilities upkeep
Total	$529.44$	100%	

### Postoperative follow-up and long-term rehabilitation

Each hospital-based follow-up consultation costs approximately $11 ± 2.2, which is notably below the typical ophthalmologist consultation fees in the Democratic Republic of the Congo, ranging from $15 to $40. The total cost of standard postoperative care per patient amounts to $77 ± 15.4 for seven scheduled visits. Consultations are conducted by a dedicated team consisting of one ophthalmologist and six ophthalmic nurses, collectively managing around 90 patients per day. Approximately 46% of children require only routine follow-up without further intervention, while the remaining 54% may need additional medical care.

### Total cost per child diagnosed and treated for bilateral cataract

The total cost per child diagnosed and successfully treated for bilateral congenital cataract in Kinshasa includes the costs of community-based screening, surgical intervention, and postoperative follow-up care. Based on the data from the RAC/AMD program and hospital cost structures, the cost per child identified through the community screening network is approximately $268.42 ± 53.7. The cost for bilateral cataract surgery and hospitalization in one session is $529.44, and the structured postoperative care, including seven follow-up visits, adds $77.00 ± 15.4 per child. This results in a total average cost of $874.86 per child, covering the full continuum from identification to long-term rehabilitation (Table 3). In this calculation, the cost for postoperative complications or procedures are not included.

**Table 3:** Total cost per child for diagnosis, treatment, and follow-up of bilateral cataract.

Cost component	Cost per child (USD)	Explanation
Community-Based Screening	$268.42 ± 53.7	Includes outreach, volunteer coordination, and staff salaries
Surgical Intervention and Hospital Stay	$529.44	Two-eye procedure with anesthesia, materials, and 7-day stay
Postoperative Follow-up (7 scheduled visits)	$77.00 ± 15.4	Structured medical care up to two years post-surgery
Total	$874.86 ± 69.1	Full process from identification to rehabilitation

### Costing of cataract management program

The cataract management program is embedded in a large-scale, community-based screening and hospital infrastructure that reaches approximately 134,400 individuals annually. This system leverages a hierarchical network of volunteers, parishes, and medical professionals, and is supported by existing healthcare institutions. From the total population screened, 38 children with bilateral cataract are identified and undergo full treatment, including preoperative evaluation, bilateral surgery in one hospital admission, and structured follow-up care for up to two years.

The total annual cost of the cataract management program is approximately $33,244 ± 2,625, which includes the cost of screening all participants and the full care continuum for identified patients. Of this total, screening activities account for $10,200 ± 2,040 (30.7%), surgical treatment and hospitalization for $20,118.72 (60.5%), and postoperative follow-up care for $2,926 ± 585 (8.8%) (Supplement Table S3).

### Simulation of program cost in a step-fixed screening model

To assess the scalability of the pediatric cataract management program, we simulated cost behavior across varying population sizes using a step-fixed cost model. This model reflects the reality of operational constraints, where a screening team covers fixed population blocks (134,400 individuals), and costs only increase when a new team is required. As the population increases, screening costs rise in steps, while treatment costs scale linearly with the number of children identified and treated (2.83 per 10,000 individuals).

**Fig. 2:**
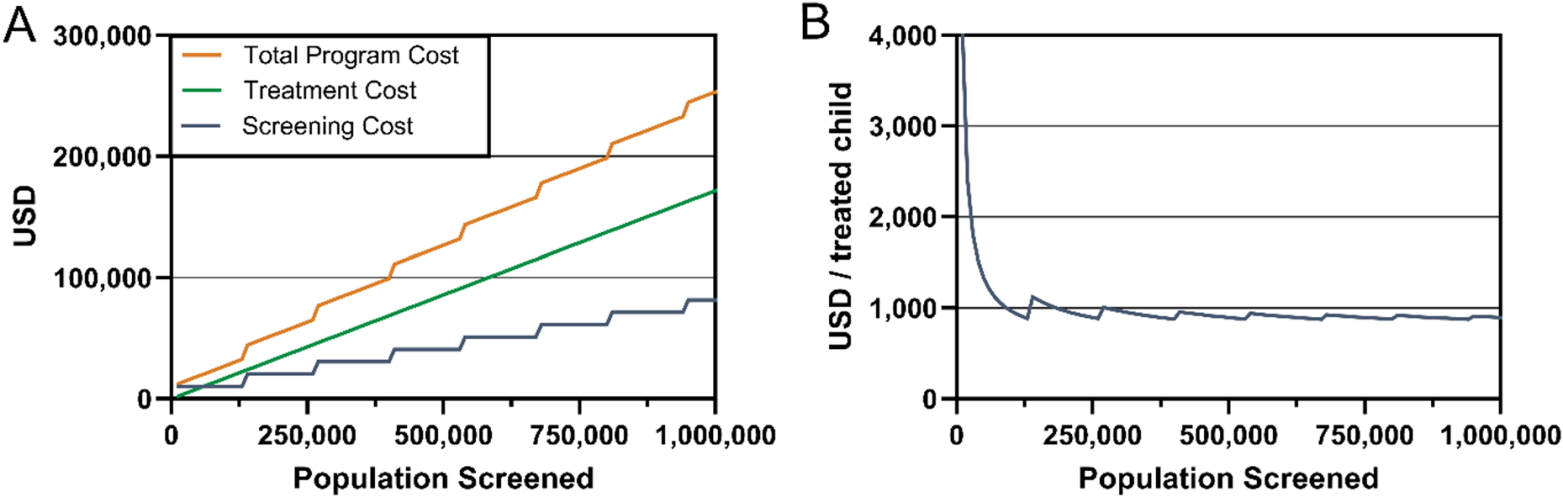
Simulation of cost structure in a step-fixed cataract screening and treatment program. (**A**) Total program cost (orange), treatment cost (green), and screening cost (blue) plotted against increasing population coverage. Screening costs rise in discrete steps, while treatment costs increase linearly. (**B**) The average cost per treated child declines rapidly at first and stabilizes around $894 as economies of scale are realized.

At a screening population of 1,000,000 individuals, the model activates eight screening blocks and identifies 283 children with bilateral cataract. Total screening costs reach $81,600 ± 16,320, and treatment costs amount to $171,622.52 ± 4,358, resulting in a total program cost of $253,222.52 ± 20,678 (Fig. 2A). The average cost per treated child stabilizes around $874.86 ± 69.1, reflecting increasing cost-efficiency as more children are identified per fixed screening investment (Fig. 2B).

## Discussion

### Summary of main findings

In this cost analysis of a pediatric cataract program in Kinshasa, we found that providing the full continuum of care, from community-based case identification through surgery and two-year follow-up, was approximately $874 per treated child. Notably, the surgical intervention (bilateral cataract surgery in one admission) accounted for the largest portion (~ 60%) of per-child costs (about $529), with the remainder due to the expansive community screening network (~ 30%) and structured postoperative follow-up (~ 10%). While the absolute cost may seem high in this low-resource context, it reflects a comprehensive approach ensuring children are found, treated, and rehabilitated. Our results underscore that pediatric cataract management is not just a one-time surgical event but a multi-step process requiring coordination across community volunteers, clinical teams, and rehabilitation services^2^.

### Comparison with previous cost studies

Relatively few studies have quantified the cost of pediatric cataract care in low-income settings, and direct comparisons must consider differences in scope. A notable study from two child eye health tertiary facilities (CEHTFs) in Malawi and Zambia reported the direct cost per child for cataract surgery (including in-hospital care) as $202 in Malawi and $277 in Zambia (2011 US dollars)^9^. These figures are substantially lower than our Kinshasa hospital cost ($529 for bilateral surgery and 7-day stay; 2024 US dollars). However, the Malawi/Zambia analysis excluded community outreach costs and long-term follow-up, focusing mainly on surgical and immediate perioperative expenses^9^. In contrast, high-volume specialized centers can achieve lower unit costs. For example, a cost analysis in India found the average provider cost of pediatric cataract surgery to be around $314–$398 in a tertiary hospital (2018–2019 US dollars)^13^. Their analysis emphasized that fixed costs (personnel, facility) are diluted by volume, and bulk procurement lowers consumable prices.

The key difference is that our cost per child includes community screening and case-finding expenditures (~$268 per child identified). Most published cost analyses^9,13^ adopt the provider/hospital perspective, starting from when a patient presents for surgery. In practice, children with cataract often do not present on their own due to low awareness and various access barriers in impoverished communities^14^. Our study intentionally captured the resources needed to identify patients. In Kinshasa, an extensive network of 20 + screening volunteers and community health workers was necessary to reach 134,400 individuals across hundreds of parishes in a year. This yielded 38 treatable bilateral cataract cases. The cost per screening was very low ($0.08 per person), but the aggregate investment in the screening program was significant, accounting for roughly one-third of total program costs. The yield of 28.3 bilateral cataract cases per 100,000 population screened is in line with the expected prevalence of childhood cataract in low- to middle-income regions^15^.

By showcasing the full process from identification to rehabilitation, we also highlight an often neglected aspect: children’s post-surgical reintegration into society^16^. The ultimate goal is not just anatomic success (clear visual axis) but that the child can see well enough to develop academically and socially^17^. Our volunteer network followed up at the community level to ensure children were using their glasses and attending school after surgery^18^. This extends beyond traditional medical care into the domain of social support and education. In our context, such engagement was facilitated by the community-based rehabilitation (CBR) program (RAC/AMD) that is part of the team. The CBR workers continued to visit the children at home, encourage adherence to patching or glasses, and liaise with schools if needed. The importance of this “last mile” cannot be overstated: without it, the impact of surgery might be lost due to amblyopia or social exclusion. We advocate that pediatric blindness initiatives in low-resource settings incorporate similar follow-up to secure the functional vision gains for each child.

### Limitations and strengths

Our costing reflects a single program and time period in Kinshasa, thus absolute figures should not be overgeneralized, as prices vary by setting, procurement routes and volume (with intraocular lens/consumables often the main cost drivers)^19^. For instance, our consumables were sometimes more expensive due to importation costs or lack of bulk purchasing power in Kinshasa. We adopted a provider perspective and did not quantify indirect patient and caregiver costs. Given that affordability and travel are dominant barriers to pediatric eye care in Africa, future work should include a societal perspective in line with CHEERS 2022 guidance^14,20–22^. The scale-up model assumed step-fixed screening with constant case-finding, in practice, the number of cases found varies by context and may decline as easier-to-reach cases are exhausted^23^. Nevertheless, to our knowledge, this is the first study from a sub-Saharan African low- and middle-income country to describe in detail the structure and methods required to build such a program and to quantify and justify the provider costs needed to deliver it. It combines hospital bills, payroll data, program records, and stakeholder interviews and, importantly, explicitly includes community-based screening and rehabilitation, which are often omitted from cost estimates.

### Implications for policy and research

Courtright et al. estimated that 15–35% of childhood blindness in Africa is due to cataract and about 19,000 new pediatric cataract cases occur annually across the continent^2^. Identifying these children is labor-intensive; effective programs in Africa have consistently relied on community engagement and key informants for case detection^10^. The vast majority of children in our study were identified through active screening days in the community, not through spontaneous presentation to hospitals^6^. We integrated a step-fixed costing model for the screening program, which can help planners understand the investment needed to expand identification efforts to a provincial or national scale. For example, covering a population of 1,000,000 (about 8 times our current base) would require ~ 8 screening teams but could identify ~ 283 children with bilateral cataract, with a program cost of $253,222 (2014 US dollars). Ministries of Health and supporting NGOs could use such data to advocate for scaling up pediatric eye care, knowing that initial investments (training volunteers, etc.) will have multiplying benefits as more children are reached.

If a region or country is aiming to eliminate childhood cataract, they must budget not just for surgeries and materials (e.g. intraocular lenses), but also for community outreach and follow-up services. These people-centred components (human outreach, health education, etc.) routinely face funding gaps, reflecting a common donor bias towards procuring equipment rather than supporting outreach and education^24^. In our setting, approximately one-third of all costs were attributable to community-based screening and follow-up activities, many of which were delivered by unpaid volunteers. These functions are essential but structurally vulnerable. Our costing underscores the need to assign explicit monetary value to this work and to progressively replace purely voluntary engagement with funded or stipended roles to support long-term sustainability.

Comprehensive pediatric cataract care in Kinshasa was feasible at $875 per child for the full continuum (identification, bilateral surgery, and structured follow-up), with surgery comprising the largest share. Although this cost may appear high in a low-resource setting, it needs to be interpreted against the lifetime benefit of restoring sight. Childhood cataract is responsible for an estimated 10 million blind-person–years globally^25^. Every child treated averts decades of blindness, yielding economic and social returns far exceeding the upfront costs. From a health economics perspective, an intervention that restores sight in a child (who may have 50–70 years of life ahead) is highly cost-effective, even if the cost is several hundred dollars^1^. This per-child estimate is also useful for communication and fundraising: a charity could state that roughly $900 can take a child from blindness to sight, including two years of care. Budgets must explicitly fund people and processes (community case-finders, case managers, follow-up coordinators) alongside consumables and equipment. Future work should include a societal perspective to capture family expenses.

## Supplementary Information

Below is the link to the electronic supplementary material.

Supplementary Material 1

## Data Availability

Aggregated cost data derived from hospital billing records, payroll information, and program expenditures are available from the corresponding author upon reasonable request.

## References

[1] Burton, M. J. et al. The *Lancet global health* commission on global eye health: vision beyond 2020. *Lancet Global Health*. **9**, e489–e551 (2021). 33607016 10.1016/S2214-109X(20)30488-5 PMC7966694

[2] Courtright, P. Childhood cataract in sub-Saharan Africa. *Saudi J. Ophthalmol.* **26**, 3–6 (2012). 23960961 10.1016/j.sjopt.2011.10.006 PMC3729567

[3] Harrabi, H., Aubin, M. J., Zunzunegui, M. V., Haddad, S. & Freeman, E. E. Visual difficulty and employment status in the world. *PLOS ONE*. **9**, e88306 (2014). 24516632 10.1371/journal.pone.0088306 PMC3917855

[4] Sonksen, P. M. & Dale, N. Visual impairment in infancy: impact on neurodevelopmental and Neurobiological processes. *Dev. Med. Child. Neurol.* **44**, 782–791 (2002). 12418621 10.1017/s0012162201002936

[5] Sheeladevi, S., Lawrenson, J. G., Fielder, A. R. & Suttle, C. M. Global prevalence of childhood cataract: a systematic review. *Eye* **30**, 1160–1169 (2016). 27518543 10.1038/eye.2016.156 PMC5023808

[6] Poschkamp, B. et al. Management of bilateral congenital and juvenile cataracts in a Low-Income country: patient Identification, treatment Outcomes, and follow up. *Children* **11**, 1064 (2024). 39334598 10.3390/children11091064 PMC11430800

[7] Mwende, J. et al. Delay in presentation to hospital for surgery for congenital and developmental cataract in Tanzania. *Br. J. Ophthalmol.* **89**, 1478–1482 (2005). 16234457 10.1136/bjo.2005.074146 PMC1772945

[8] Sen, P. et al. Causes of delayed presentation of pediatric cataract: A questionnaire-based prospective study at a tertiary eye care center in central rural India. *Indian J. Ophthalmol.* **68**, 603–607 (2020). 32174578 10.4103/ijo.IJO_872_19 PMC7210835

[9] Evans, C. T. et al. A cost analysis of pediatric cataract surgery at two child eye health tertiary facilities in Africa. *J. AAPOS*. **18**, 559–562 (2014). 25454021 10.1016/j.jaapos.2014.08.005 PMC4268264

[10] Kilangalanga, J. N. et al. Role of a Community-based program for identification and referral of pediatric cataract patients in Kinshasa, Democratic Republic of the congo. *Middle East. Afr. J. Ophthalmol.* **26**, 83–88 (2019). 31543665 10.4103/meajo.MEAJO_273_18 PMC6737789

[11] Bowman, R. J. C. How should blindness in children be managed? *Eye (Lond)*. **19**, 1037–1043 (2005). 16304582 10.1038/sj.eye.6701988

[12] Roberts, R. R., Mensah, E. K. & Weinstein, R. A. A guide to interpreting economic studies in infectious diseases. *Clin. Microbiol. Infect.* **16**, 1713–1720 (2010). 20825433 10.1111/j.1469-0691.2010.03366.x

[13] Kapse, R. R. et al. Cost analysis of pediatric cataract surgery in a tertiary eye care hospital in Western India. *Indian J. Ophthalmol.* **70**, 420–424 (2022). 35086208 10.4103/ijo.IJO_1229_21 PMC9023949

[14] Alrasheed, S. H. A systemic review of barriers to accessing paediatric eye care services in African countries. *Afr. Health Sci.* **21**, 1887–1897 (2021). 35283961 10.4314/ahs.v21i4.47 PMC8889803

[15] Khokhar, S. K. et al. Pediatric cataract. *Indian J. Ophthalmol.* **65**, 1340 (2017). 29208814 10.4103/ijo.IJO_1023_17 PMC5742962

[16] Frech, S. et al. Educational and medical aspects after cataract surgery of bilaterally blind children in Kinshasa—Perception of parents and children. *Child. (Basel)*. **9**, 1683 (2022). 10.3390/children9111683 PMC9688321 36360411

[17] Ganesh, S., Sethi, S., Srivastav, S., Chaudhary, A. & Arora, P. Impact of low vision rehabilitation on functional vision performance of children with visual impairment. *Oman J. Ophthalmol.* **6**, 170–174 (2013). 24379551 10.4103/0974-620X.122271 PMC3872566

[18] Lambert, S. R., DuBois, L., Cotsonis, G., Hartmann, E. E. & Drews-Botsch, C. Spectacle adherence among four-year-old children in the infant aphakia treatment study. *Am. J. Ophthalmol.* **200**, 26–33 (2019). 30633891 10.1016/j.ajo.2018.12.017 PMC6445735

[19] Gogate, P., Dole, K., Ranade, S. & Deshpande, M. Cost of pediatric cataract surgery in Maharashtra, India. *Int. J. Ophthalmol.* **3**, 182–186 (2010). 22553549 10.3980/j.issn.2222-3959.2010.02.22 PMC3340774

[20] Bronsard, A., Geneau, R., Shirima, S., Courtright, P. & Mwende, J. Why are children brought late for cataract surgery? Qualitative findings from Tanzania. *Ophthalmic Epidemiol.* **15**, 383–388 (2008). 19065431 10.1080/09286580802488624

[21] Husereau, D. et al. Consolidated health economic evaluation reporting standards 2022 (CHEERS 2022) statement: updated reporting guidance for health economic evaluations. *Value Health*. **25**, 3–9 (2022). 35031096 10.1016/j.jval.2021.11.1351

[22] Schulz, H. et al. Predictors of attendance at the first follow-up and poor visual outcome after paediatric cataract surgery in Kinshasa for the years 2001–2021. *Trop. Med. Health*. **53**, 32 (2025). 40001188 10.1186/s41182-025-00706-8 PMC11863572

[23] Duke, R. et al. Using key informants to estimate prevalence of severe visual impairment and blindness in children in cross river State, Nigeria. *J. AAPOS*. **17**, 381–384 (2013). 23911130 10.1016/j.jaapos.2013.05.004

[24] Tsai, F. J., Lee, H. & Fan, V. Y. Perspective and investments in health system strengthening of Gavi, the vaccine alliance: a content analysis of health system strengthening-specific funding. *Int. Health*. **8**, 246–252 (2016). 26612851 10.1093/inthealth/ihv063 PMC6281386

[25] Shamanna, B. & Muralikrishnan, R. Childhood cataract: Magnitude, Management, economics and impact. *Community Eye Health*. **17**, 17–18 (2004). 17491800 PMC1705721

