# Supplementary material for: Provider-perspective cost analysis of comprehensive pediatric cataract management in a low-income setting
Source: Sci Rep. 2026 Jan 19;16:2641. doi: 10.1038/s41598-025-32417-9 (PMC12823557; doi:10.1038/s41598-025-32417-9)
Supplement: Supplementary file 1 — Supplementary Material 1 [file 41598_2025_32417_MOESM1_ESM.docx]

# Supplement

## Cheers-Checklist

To ensure transparency and reproducibility, this study was assessed against the Consolidated Health Economic Evaluation Reporting Standards (CHEERS 2022) checklist [1]. The completed checklist (**Supplement table 1**) indicates where each item is addressed within the manuscript. As this work represents a cost analysis rather than a full economic evaluation with health outcomes, several items (e.g., outcome measurement/valuation, comparators) are not applicable and are noted as such.

**Supplement table 1: Cheers 2022 Checklist**

| **Section/topic** | **Item no.** | **Guidance for reporting** | **Reported in section** |
| --- | --- | --- | --- |
| **Title** | 1 | Identify the study as an economic evaluation and specify the interventions being compared. | Abstract (cost analysis stated, no comparators) |
| **Abstract** | 2 | Structured summary with context, key methods, results, alternatives. | Abstract |
| **Introduction: Background and objectives** | 3 | Context, question, practical relevance. | Introduction |
| **Methods: Health economic analysis plan** | 4 | Whether a HEAP was developed/where available. | A pre-specified standalone HEAP was not developed; however, the Methods section outlines the analytic plan in detail, including perspective, population, data sources, cost categories, currency/year, modeling assumptions, and simulation approach. |
| **Study population** | 5 | Characteristics of study population. | Methods (children with bilateral cataract in Kinshasa; program-level) |
| **Setting and location** | 6 | Context influencing findings. | Introduction; Methods (Kinshasa, DRC) |
| **Comparators** | 7 | Interventions/strategies compared and why. | Not applicable (single-arm program cost analysis; no comparator) |
| **Perspective** | 8 | Perspective(s) and rationale. | Methods (provider perspective) |
| **Time horizon** | 9 | Time horizon and justification. | Costs were measured at a single time point (2024 USD), but the analytic pathway explicitly covered the full continuum of care up to two years after surgery, including scheduled follow-up and rehabilitation. The two-year interval was chosen because it reflects the standard clinical rehabilitation course for pediatric cataract in this setting. |
| **Discount rate** | 10 | Discount rate(s) and reason. | Not applied (short horizon; all costs 2024 USD) |
| **Selection of outcomes** | 11 | Benefit/harm measures. | Not applicable (cost analysis only; no health outcomes like QALYs) |
| **Measurement of outcomes** | 12 | How outcomes were measured. | Not applicable |
| **Valuation of outcomes** | 13 | Population/methods used to value outcomes. | Not applicable |
| **Measurement & valuation of resources and costs** | 14 | How costs were valued. | Methods (hospital bills, payroll, interviews; cross-checks) |
| **Currency, price date, and conversion** | 15 | Dates, currency, year. | Methods (2024 USD) |
| **Rationale and description of model** | 16 | Modeling detail/why used; public availability. | Methods (step-fixed costing simulation in Python; public availability on request) |
| **Analytics and assumptions** | 17 | Data transforms, extrapolation, validation. | Methods (team capacity blocks; linear treatment scaling); Discussion–Limitations (generalizability). |
| **Characterizing heterogeneity** | 18 | Subgroup variation methods. | There are no subgroups. |
| **Characterizing distributional effects** | 19 | Distributional/priority-population adjustments. | Not applicable |
| **Characterizing uncertainty** | 20 | Methods to characterize uncertainty. | Methods (±20% variation of staff salaries and follow-up unit costs); Discussion – Limitations (contextual variability noted) |
| **Engagement with patients/others** | 21 | Engagement approaches. | Methods (stakeholder interviews; community/parish network) |
| **Results: Study parameters** | 22 | Analytic inputs (values, ranges, distributions). | Results,  Tables 1–3, Supplement table 2 (point estimates; no ranges/distributions) |
| **Summary of main results** | 23 | Mean values for costs/outcomes; overall measure. | Abstract; Results; Discussion |
| **Effect of uncertainty** | 24 | How uncertainty affects findings; discount/time horizon effects. | Results (reported ranges for key unit costs); Discussion – Limitations (price/procurement variability) |
| **Effect of engagement** | 25 | Impact of stakeholder involvement. | Results; Discussion (community engagement central to identification/adherence) |
| **Discussion: Findings, limitations, generalizability, current knowledge** | 26 | Key findings, limitations, ethics/equity and implications. | Discussion: Summary of main findings, limitations and strengths, Implications for policy and research |
| **Source of funding** | 27 | Funding and funder role. | Funding section |
| **Conflicts of interest** | 28 | Authors’ COI | Conflict of interest section |

## Process Diagram

The process of the Pediatric Cataract Surgery Management Program (**Supplement figure 1**) begins with conceptualizing the juvenile cataract program and recruiting local volunteers who are trained not only in identifying potential cases but also in understanding local circumstances, basic eye health concepts, and communication with families. These trained volunteers travel into various districts and communities, often remote or underserved, to screen children for signs of vision impairment. Once a child is identified, they are referred to an ophthalmologist who evaluates their condition and confirms eligibility for the program, ensuring that standard cataract surgery is appropriate for their case.

After eligibility is established, the child proceeds to a designated surgical center, where administrative and planning tasks take place. The travel usually by public transport and organized privately by the family, and if not possible by the screening volunteer, who picks up the child and brings it to the hospital. During pre-operative assessments, the child meets with both a pediatrician and an anesthetist to verify that there are no underlying health issues that would complicate anesthesia or the surgery itself. If the child is sufficiently healthy for the surgery, the operation is scheduled, and the family and case workers arranges for the child’s travel and arrival at the hospital on the appointed date.

The hospital phase involves admission, thorough pre-operative checks to confirm patient details and surgical targets, and then the cataract surgery itself. Following the procedure, the child recovers initially in a dedicated recovery room under close observation, and then transitions to a normal hospital ward for continued monitoring. Once stable and cleared by medical staff, the child is discharged, often accompanied by guidance for immediate post-operative care.

Over the subsequent two years, the child returns periodically for follow-up examinations at recommended intervals. These follow-ups help detect any potential complications, such as secondary cataracts, and ensure timely intervention. They also allow for adjustments in therapy, prescription of glasses, or additional support as the child’s vision stabilizes. Ultimately, the program extends beyond clinical interventions: volunteers revisit the child’s home environment and community setting. This final stage evaluates not just medical success, but also educational attendance, proper use of prescribed glasses, and developmental milestones, supporting the child’s reintegration into everyday life and ensuring that restored vision translates into tangible improvements in their quality of life.


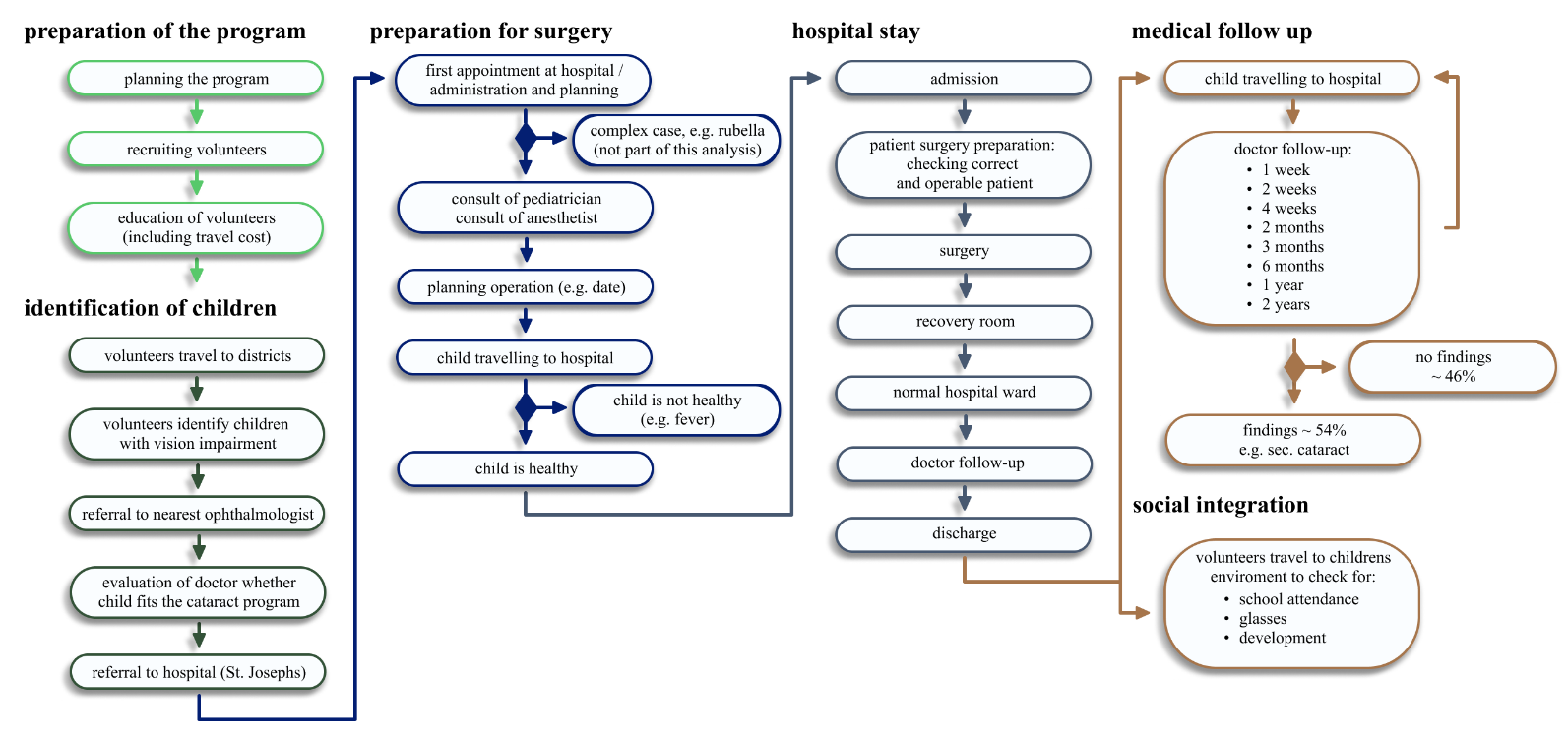


Supplement figure 1: Process diagram

## Cost analysis of Community-based screening program

The personal cost of the community-based screening program is presented (Supplement table 2).

**Supplement table 2: Cost Analysis of Community-Based Screening Program for Pediatric Cataract in Kinshasa (RAC/AMD)**

| Component | Quantity | Cost per Month (USD) | Annual Cost (USD) |
| --- | --- | --- | --- |
| Ophthalmic Nursing Staff | 1 person | $350±70 | $4,200±840 |
| Case Manager | 1 person | $250±50 | $3,000±600 |
| Social Worker | 1 person | $250±50 | $3,000±600 |
| Screening Volunteers | 20 | $0 | $0 |
| Screening Volunteers | 12 | $0 | $0 |
| Total Annual Cost | | | $10,200±2,040 |

## Community-based screening structure


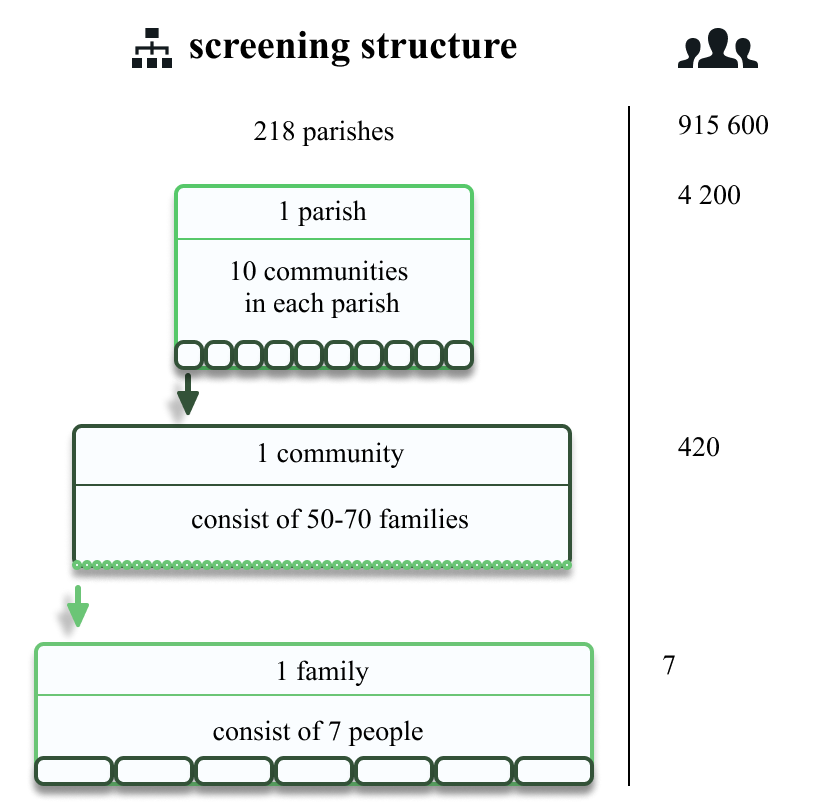
Parishes act as local hubs in a hierarchical, community-embedded network, linking trained volunteers to communities and families to coordinate awareness, case-finding, referral to ophthalmology services, and follow-up support.

Supplement figure 2: Screening infrastructure

## Total Annual Cost of Cataract Management program

The annual program cost was $33,244.72, with community-based screening $10,200±2,040 (30.7%), surgery and hospital stay $20,118.72 (60.5%), and postoperative follow-up care $2,926.00±585.2 (8.8%).

**Supplement table 3: Total Annual Cost of Cataract Management Program (38 Children Treated)**

| Program Component | Cost per Unit | Quantity | Total Cost (USD) | Share of Total Cost |
| --- | --- | --- | --- | --- |
| Community-Based Screening | $10,200 | 134,400/Team | $10,200±2,040 | 30.7% |
| Surgery and Hospital Stay | $529.44 | 38 children | $20,118.72 | 60.5% |
| Postoperative Follow-up Care | $77.00 | 38 children | $2,926.00±585.2 | 8.8% |
| Total Program Cost |  |  | $33,244.72±2,625.2 | 100% |

## List of materials for surgery

The surgical materials typically include sterile gowns, eye drape, povidone scrub solution, povidone dermal solution, sterile gauze swab, sterile gloves, Q-Tips, viscoelastic solution (methyl cellulose or sodium hyaluronate), infusion set, syringes (1 cc, 5 cc, 10 cc), sutures 10/0 dacron, trypan blue, BSS (Lactate Ringer 500 ml), surgical blades (crescent 2.5 mm; Keratotome 3.2 mm; Sideport 15°), lidocaine 2% (subconjunctival), lidocaine 2% (intracameral), moxifloxacin 0.5% preservative-free (intracameral) and gentamicin, proparacaine eye drops, adrenaline 1 mg/ml injection vial, Tobradex eye drops (post-surgery), Diamox 250 mg tablet (one after surgery) which all together account for around 50$, and either an ordinary IOL (7–10$) or an exclusive IOL (16$).

## Interview Guides

**A: Questions for Parents / Patients with Cataract**

1. **Medical Process Questions:**
2. Who identified the visual problem first?
3. What tests were done to identify the cataract?
   1. Was there a test where a flashlight was used?
   2. Was there a test where a visual acuity chart (a chart with numbers) was used?
   3. Was there a test where a person looked through a device (e.g. Slit lamp)?
4. Was there a examination from a doctor / optometrist before the surgery was scheduled?
5. How many times did you come before the surgery was done?
6. How long did you stay in the clinic for the cataract surgery? (hours, days)
7. How many times did you come to an examination after surgery?
8. **Cost Questions:**
9. Did you have to pay for your transportation or your travel to the clinic / examinations?
   1. If Yes, how much did you pay?
   2. No
10. How do you usually travel to the appointments?
    1. on foot
    2. by bus
    3. by scooter / motorbike
    4. by car
    5. by bicycle
    6. other:
11. Did you eat while you were in the hospital?
    1. If yes: Did you have to pay for it and how much?
    2. No.
12. Did you stay somewhere that wasn’t your home? Did you pay for it and how much? Or who did it?
13. Do you know other places in Kinshasa where they perform cataract surgery?
14. Do you know the cost of a cataract surgery other people paid?
15. How much did you have to pay for a cataract surgery?
16. Do you know how much a night's stay in the hospital costs?
    1. If yes: How much did you have to pay?
    2. No.
17. How much did you pay for glasses?
18. How often do you have to get new glasses?

**B: Questions for Cataract Screeners / volunteers**

1. **Training Questions:**
2. How did you find out about this job?
   1. friends / family
   2. Online
   3. From Church
   4. Other:
3. How many people are in your training class? (e.g. 3 groups a 50 people each)
4. How many people of your training class started with you and how many will finish it?
5. How long is the training exactly (in months, years)?
6. How many training sessions must you complete before you can independently screen patients?
7. Do you learn how to perform a refraction or a retinoscopy?
8. **Screening Questions:**
9. How do you usually travel to the screening locations?
   1. on foot
   2. by bus
   3. by scooter / motorbike
   4. by car
   5. by bicycle
   6. other:
10. Are these your own means of transport?
11. Where do you perform screenings?
    1. School
    2. Childcare / kindergarten
    3. At home
    4. Other:
12. How often do you conduct screenings (per week, per month)?
13. How many children do you screen daily (a), and how many of them have to be further examined(b)?
14. How many hours do you screen on a screening day?
15. What equipment do you need for screening (e.g., flashlights, magnifying glasses, vision charts)?
16. What screening methods do you use?
    1. Visual Acuity Chart
    2. LEA Symbols
    3. Cover-Test
    4. Brueckner Test
    5. Flashlight
    6. Handheld slit lamp
    7. Refraction test
    8. Other:
17. Do you look for cataracts only or other eye issues too?
18. What happens with the children after you identified them?
19. Do you also look for children after they completed the surgery?
20. **Compensation Questions:**
21. Do you get paid?
    1. If yes, how much?
    2. No.
22. Do you get something for work, like:
    1. food
    2. accommodation
    3. transportation
    4. access to medical care
    5. Other:

**C: Questions for Trainers**

1. **Training Program Questions:**
2. How long is the training period?
3. How many people are in a training class? (e.g. 3 groups a 50 people each)
4. Do the students have to pay for the training?
5. Are provisions such as lunch, accommodations, or access to medical care provided to the trainees?
6. **Training Questions:**
7. How many participants start a course and how many complete it?
8. Where (at which location) is the training conducted?
9. What materials are needed for the course, and what is the cost of these materials?
   - From the students' perspective
   - From the teacher's perspective
10. **Questions About Trainers:**
11. How are trainers recruited?
12. How many trainers are there, and how are they recruited?
13. What is the salary for trainers?

**D: Questions for Case Managers**

1. **Patient Care Questions:**
2. How do you first learn about a patient?
3. What needs to be organized before the patient arrives at the hospital? Is this flow chart correct?
4. How many children are typically in the program and being processed at the same time?
5. How many children are typically added to the program in a month?
6. How often do appointments need to be rescheduled?
7. How do you plan the appointments (e.g., phone numbers, etc.)?
8. Do you organize transportation to and from the hospital?
9. Are the costs for transportation covered?
10. How many parents do not respond, resulting in children not being further examined?
11. How long does a patient typically stay in the hospital, or can some children be discharged immediately?
12. How often do children arrive at the hospital and cannot be operated on the same day (e.g., due to fever)?
13. How many of the identified children undergo surgery?
14. **General Questions:**
15. How much does a standard cataract surgery typically cost at your hospital?
16. How much does an overnight stay typically cost at your hospital?
17. How many staff members are there organizing the cases?
18. How many hours do you typically work per day?
19. Are costs billed to the patient?
20. Is accommodation or similar provided for you?
21. Do you have access to healthcare services? Is it at a reduced or higher cost?
22. What is your salary?

**E: Questions for the Program Manager**

1. **Medical Services Questions:**
2. What is the cost of adult cataract surgery (not pediatric cataract) at the participating hospital for patients?
3. Do you know the cost of adult cataract surgery at other hospitals in Kinshasa?
4. What is the cost of a pediatric cataract surgery (not adult cataract) at the participating hospital for patients?
5. Do you know the cost of pediatric cataract surgery at other hospitals in Kinshasa?
6. What is the cost for patients for a minor / major surgery at the hospital?
7. Do you know the cost of an intraocular lens and where do you order it?
8. What is the cost of an overnight stay at the hospital?
9. What is the cost for patients for standard outpatient treatment?
10. How long do children typically stay in the hospital?
11. What do you think causes the most costs for cataract surgery in children?
12. **Staffing Questions:**
13. How many trainers are involved in this program? Do you know their salary? If not, what do you estimate?
14. How many cataract finders are involved in this program? Do you know their salary? If not, what do you estimate?
15. How many case managers are involved in this program? Do you know their salary? If not, what do you estimate?
16. How many doctors are involved in this program? Do you know their salary? If not, what do you estimate?
17. How many optometrists are involved in this program? Do you know their salary? If not, what do you estimate?
18. How many doctors or optometrists are involved in postoperative care? Do you know their salary? If not, what do you estimate?

**F: Questions for Doctors**

1. **Patient Care Questions:**
2. How many children do you examine to plan a surgery per week / month?
3. Estimate which percentage of children with suspicion of cataract get also a surgery?
4. How often do children have to be sent home without surgery and the operation postponed (e.g., due to fever)?
5. How long do children typically stay in the hospital post-surgery?
6. Where are the lenses for the operation sourced from?
7. How much does a lens for the operation cost?
8. How long does a cataract surgery for children take?
9. What percentage of children need to be seen by another specialty department (e.g., Pediatrics) in addition to the surgery?
10. How often are blood samples taken before cataract surgery and what is getting tested (e.g. blood count)?
11. How many follow-up examinations after surgery do you conduct?
12. **Cost Factors Questions:**
13. Do you know the cost of adult cataract surgery (not pediatric cataract) at St. Joseph’s hospital for patients?
14. Do you know the cost of adult cataract surgery at other hospitals in Kinshasa?
15. Do you know the cost of a pediatric cataract surgery (not adult cataract) at the participating hospital for patients?
16. Do you know the cost of pediatric cataract surgery at other hospitals in Kinshasa?
17. How much does an overnight stay in the hospital cost?
18. What is the cost of anesthesia for a child?
19. How much does a standard lens cost?
20. Where do the lenses come from?
21. How have children to pay for glasses?
22. How often do children have to get new glasses?

## Literature

1 Husereau D, Drummond M, Augustovski F, *et al.* Consolidated Health Economic Evaluation Reporting Standards 2022 (CHEERS 2022) Statement: Updated Reporting Guidance for Health Economic Evaluations. *Value Health*. 2022;25:3–9. doi: 10.1016/j.jval.2021.11.1351
